# Fatal stroke with acute simultaneous bilateral common carotid artery occlusion presenting as sudden coma: A case report

**DOI:** 10.1016/j.radcr.2023.11.010

**Published:** 2023-11-24

**Authors:** Yuki Koike, Hideki Endo, Tomoki Fuchizaki, Kenji Kamiyama, Toshiaki Osato, Hirohiko Nakamura

**Affiliations:** Department of Neurosurgery, Nakamura Memorial Hospital, South 1, West 14, Chuo-ku, Sapporo, Hokkaido 060-8570, Japan

**Keywords:** Bilateral common carotid artery occlusion, Cerebral infarction, Coma, Common carotid artery, Magnetic resonance angiography, Magnetic resonance imaging

## Abstract

Common carotid artery occlusion is rare. Bilateral common carotid artery occlusion is extremely rare and, to our knowledge, has hardly ever been reported. This report describes a case of fatal stroke with acute simultaneous bilateral common carotid artery occlusion presenting as sudden coma. A 90-year-old woman was transferred to our hospital by ambulance with a sudden coma. She had a history of atrial fibrillation but had not taken any oral antithrombotic medication in recent years. She had been receiving house calls for dehydration in the previous week. Magnetic resonance imaging showed extensive cerebral infarcts in both cerebral hemispheres, and magnetic resonance angiography revealed bilateral common carotid artery occlusion. Acute recanalization therapy was not performed because of the extensive cerebral infarction, the patient's advanced age, and her poor ability to perform activities of daily living. On the day after onset, she died of massive cerebral infarction and marked brain swelling.

## Introduction

Common carotid artery (CCA) occlusion is rare, accounting for less than 1% of all strokes [1]. Bilateral CCA occlusion is even rarer, and, to our knowledge, has been the subject of few reports [1], [2]. In this report, we describe a case of fatal stroke with acute simultaneous bilateral CCA occlusion presenting as sudden coma.

## Case report

**Fig. 1:**
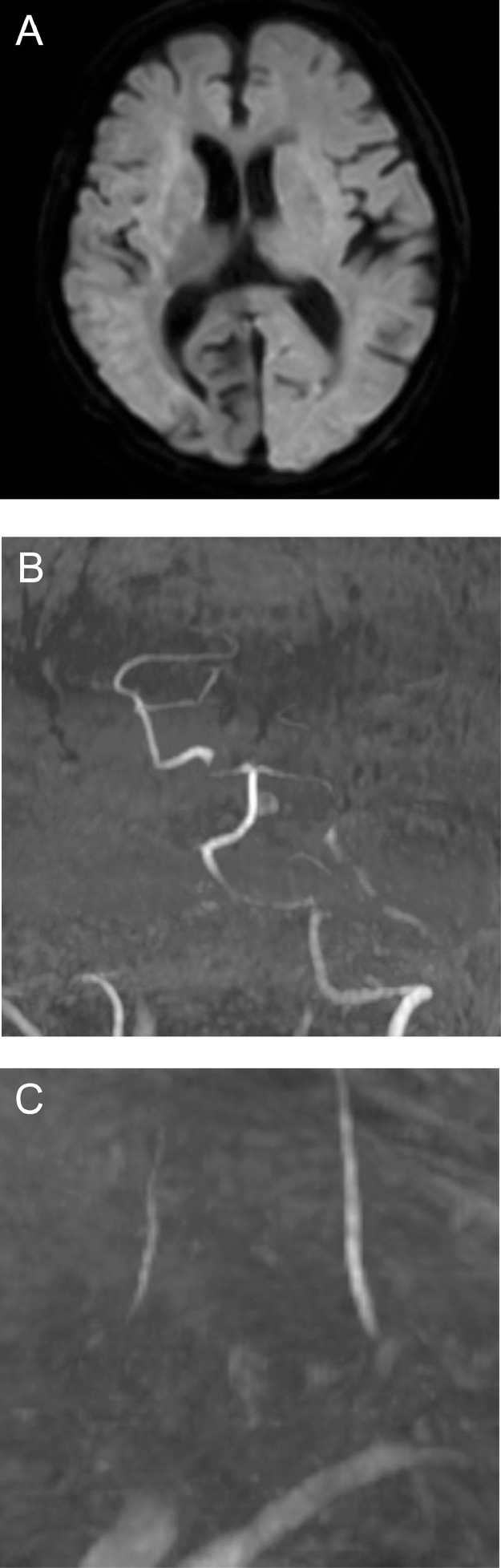
Magnetic resonance (diffusion-weighted) images obtained at admission showing extensive acute infarcts in both cerebral hemispheres (A) Magnetic resonance angiographic images (B, intracranial; C, neck) revealing bilateral common carotid artery occlusion.

**Fig. 2:**
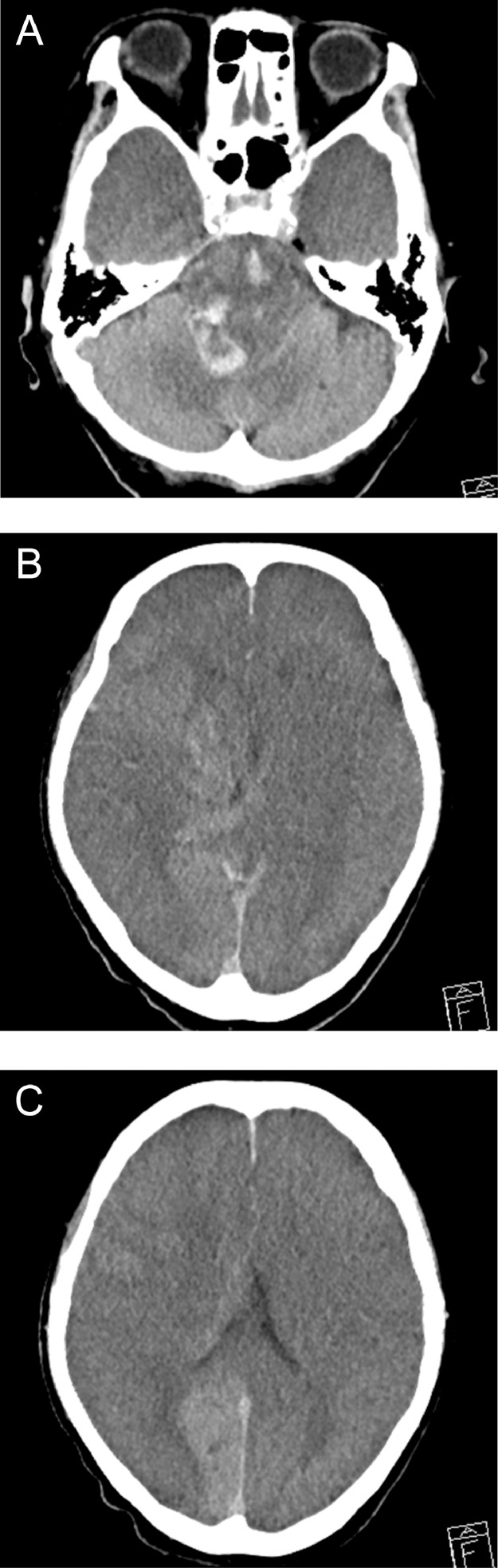
Computed tomography scan obtained on the day after onset demonstrating massive cerebral infarcts and marked brain swelling.

The case was a 90-year-old woman who had been receiving house calls for dehydration during the previous week and was transported by ambulance to our hospital after the sudden onset of a coma. She had a medical history of hypertension, diabetes, atrial fibrillation, congestive heart failure, and Parkinson's disease and needed assistance with activities of daily living (modified Rankin Scale score, 4). She had taken antithrombotic medications in the past but not in recent years. Upon emergency arrival, she had severe neurological deficits, including deep coma (Glasgow Coma Scale, 4; E1, V1, M2) and tetraplegia (National Institutes of Health Stroke Scale score, 30) but no unilateral symptoms, such as conjugate deviation or hemiparesis. Magnetic resonance imaging at 105 minutes after onset revealed extensive infarcts in both cerebral hemispheres, with only the right posterior cerebral artery territory being spared (Fig. 1A). Magnetic resonance angiography showed arterial signals in the vertebrobasilar system (posterior circulation) and none in the entire anterior circulation, including the CCA bilaterally (Figs. 1B and C). We diagnosed acute cerebral infarction caused by bilateral CCA occlusion. We did not perform acute recanalization therapy because of the extensive cerebral infarction, the patient's advanced age, and her limited ability to perform activities of daily living. On the day after onset, computed tomography demonstrated massive cerebral infarction and marked brain swelling (Fig. 2). The patient died on the same day.

## Discussion

We have encountered an extremely rare case of acute simultaneous bilateral CCA occlusion that resulted in a fatal stroke. Occlusion of the CCA is rare, and bilateral CCA occlusion is even rarer [1]. A previous review summarized 7 cases of bilateral CCA occlusion, all of which were symptomatic [1]. Five of those cases were attributed to atherosclerosis and the remaining 2 were complications after irradiation [1]. Therefore, we speculate that the lesions in those 7 cases had longer clinical courses, progressing slowly and not presenting as sudden acute occlusions. Unlike those cases, our patient had acute simultaneous bilateral CCA occlusion associated with cardiogenic embolism. To the best of our knowledge, there have been no similar reports in the past. This report provides evidence that acute bilateral occlusion can occur in the CCA.

Our patient presented with a sudden coma. Coma is one of the most common symptoms of onset of ischemia in the posterior circulation. Anterior circulation ischemia often presents with unilateral cerebral hemispheric symptoms, but bilateral occlusion of the internal carotid artery (ICA) has also been reported to cause coma, albeit rarely [3], [4]. Bilateral ICA occlusion may cause critical cerebral ischemia with limited collateral pathways. Bilateral CCA occlusion may further limit collateral flow through the external carotid artery. Therefore, acute bilateral CCA occlusion could lead to more severe and irreversible cerebral infarction earlier, as in our case (Figs. 1 and 2). Acute recanalization therapy (ie, mechanical thrombectomy) has been used for acute bilateral ICA occlusion [4], [5], [6], [7], but the therapeutic time window may be even shorter for acute bilateral CCA occlusion. Prompt and appropriate diagnostic imaging is required to provide the best possible acute treatment. In the present case, magnetic resonance imaging/angiography was useful for diagnostic purposes (Fig. 1).

Furthermore, a case with bilateral CCA occlusion would have a large volume of causative thrombus. In our patient, the following 2 points may have played an important role in thrombus formation: first, the patient was not taking anticoagulants despite having atrial fibrillation and congestive heart failure; second, she had been dehydrated for about a week before onset. A case of extensive thromboembolism of the innominate trunk, right subclavian artery, and bilateral CCA, as well as pulmonary embolism, has been reported [2]. We recommend investigation for concomitant embolism in other organs when bilateral CCA occlusion is encountered.

## Conclusions

This report describes a rare case of fatal stroke with acute simultaneous bilateral CCA occlusion that presented as sudden coma. Acute simultaneous bilateral CCA occlusion is extremely rare but can occur. Bilateral anterior circulation stroke presenting with sudden coma is rare, but indicates critical cerebral ischemia and requires prompt and appropriate imaging.

## Ethical statement

All procedures performed in studies involving human participants were in accordance with the ethical standards of the institution and/or national research committee and with the 1964 Helsinki declaration and its later amendments or comparable ethical standards. The study was approved by the Ethics Committee of Nakamura Memorial Hospital (No. 2023102601).

## Patient consent

Written informed consent was obtained from the patient's daughter.

## CRediT authorship contribution statement

**Yuki Koike:** Validation, Writing – review & editing. **Hideki Endo:** Conceptualization, Methodology, Validation, Formal analysis, Investigation, Resources, Data curation, Writing – original draft, Writing – review & editing, Visualization, Project administration. **Tomoki Fuchizaki:** Validation, Formal analysis, Investigation, Resources, Writing – review & editing. **Kenji Kamiyama:** Supervision. **Toshiaki Osato:** Supervision. **Hirohiko Nakamura:** Supervision.
